# Interdisciplinary Periodontal and Prosthetic Considerations in a Patient With Amelogenesis Imperfecta: A Case Report

**DOI:** 10.1002/ccr3.73025

**Published:** 2026-06-25

**Authors:** Kubra Burcu Yildirim, Gizem Ince Kuka, Hare Gursoy

**Affiliations:** ^1^ Department of Advanced Education in General Dentistry Nova Southeastern University College of Dental Medicine Fort Lauderdale Florida USA; ^2^ Department of Periodontology Yeditepe University Faculty of Dentistry Istanbul Türkiye; ^3^ Department of Periodontology, Hamidiye Faculty of Dentistry University of Health Sciences Istanbul Türkiye

**Keywords:** amelogenesis imperfecta, case report, crown lengthening, multidisciplinary treatment, periodontal surgery, prosthetic rehabilitation

## Abstract

Amelogenesis imperfecta (AI) comprises a clinically and genetically heterogeneous group of conditions characterized by enamel hypoplasia and/or hypomineralization, which frequently complicate functional rehabilitation and restorative treatment planning in affected patients. This case report describes the interdisciplinary management of a young male patient diagnosed with amelogenesis imperfecta. Following comprehensive initial periodontal therapy, a site‐specific crown lengthening approach was performed to re‐establish adequate clinical crown height and biologic width, thereby enabling subsequent prosthetic rehabilitation with ceramic crowns. Clinical and functional outcomes remained stable during a 1‐year follow‐up period. This case highlights the importance of early interdisciplinary treatment planning in patients with amelogenesis imperfecta to prevent further hard and soft tissue loss, facilitate predictable prosthetic rehabilitation, and support functional outcomes and oral health–related quality of life.

**Key Clinical Message**

Amelogenesis imperfecta promotes plaque retention, predisposing patients to gingival inflammation, pocket deepening, and fibrotic tissue changes. Periodontal stabilization—through non‐surgical therapy and site‐specific surgical crown lengthening limited to sites with prosthetic need—is a prerequisite for rehabilitation. In young patients, early comprehensive treatment planning prevents progressive hard and soft tissue deterioration.

## 1 Introduction

Amelogenesis imperfecta (AI) describes a clinically and genetically diverse group of conditions affecting tooth enamel through hypoplasia and/or hypomineralization [1, 2, 3]. Consequently, enamel may present as soft, thin, fragile, pitted, flaked, or discolored [1]. Although AI primarily involves enamel formation, it can significantly impair oral health–related quality of life (OHRQoL) by complicating mastication, hindering oral hygiene, causing dental hypersensitivity, and, most importantly, leading to esthetic concerns that negatively affect self‐esteem and psychosocial well‐being [4, 5].

The prevalence of AI varies widely among populations, with reported rates ranging from approximately 1 in 700 to 1 in 14,000 individuals [1, 2, 6, 7]. AI represents a group of inherited developmental enamel disorders that typically affect most, if not all, teeth and may occasionally be associated with morphological or biochemical alterations in other anatomical structures [3]. Depending on the underlying genetic and clinical characteristics, AI is broadly classified into hypoplastic, hypomaturation, hypocalcified, or mixed types [2]. Regardless of subtype, the management of AI is often complex, prolonged, and financially demanding, and optimal outcomes are most commonly achieved through a multidisciplinary treatment approach [8, 9].

Full‐mouth restorative rehabilitation is widely regarded as the gold standard treatment for patients with AI, as it addresses esthetic deficiencies, reduces or eliminates hypersensitivity, restores function, and contributes to the long‐term preservation of the dentition [10, 11]. However, several clinical challenges complicate restorative management in AI patients, including insufficient clinical crown height resulting from enamel loss and a reduced vertical dimension. Limited crown height decreases the available surface area for tooth preparation, which in turn compromises restoration retention and resistance form [12, 13, 14]. Indeed, loss of retention has been identified as one of the primary causes of crown failure in prosthetic dentistry [15].

This case report presents the multidisciplinary management of a young patient diagnosed with amelogenesis imperfecta, highlighting the importance of comprehensive periodontal therapy and crown lengthening procedures to address insufficient crown height, followed by definitive restorative rehabilitation.

## 2 Case Presentation

### 2.1 Patient Information and Chief Complaint

A 20‐year‐old male patient was referred to the Department of Periodontology, Yeditepe University Faculty of Dentistry. His chief complaints were gingival bleeding, tooth hypersensitivity, and an unaesthetic dental appearance.

### 2.2 Clinical and Radiographic Findings

**FIGURE 1:**
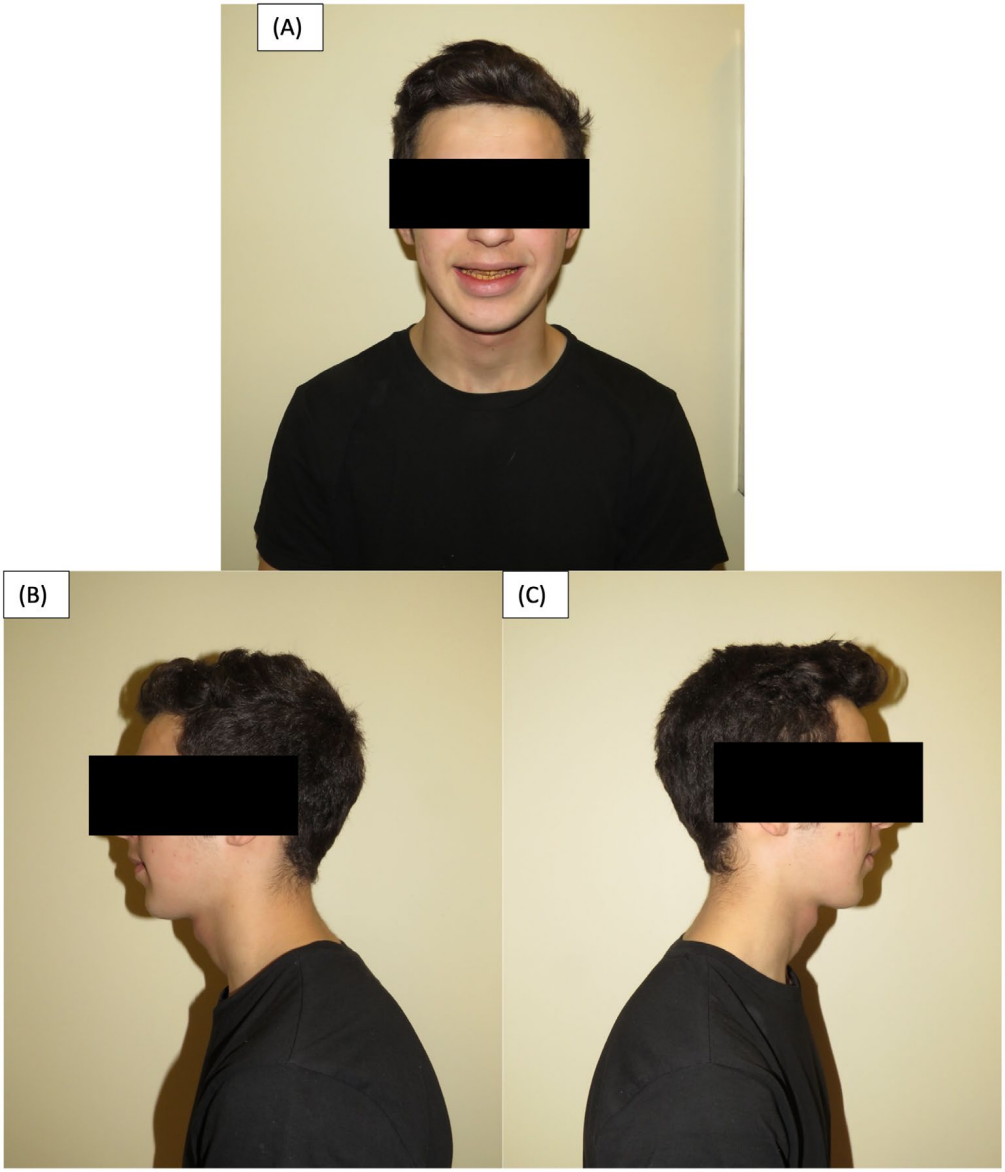
(A–C) Pre‐operative frontal and lateral extra‐oral view.

**FIGURE 2:**
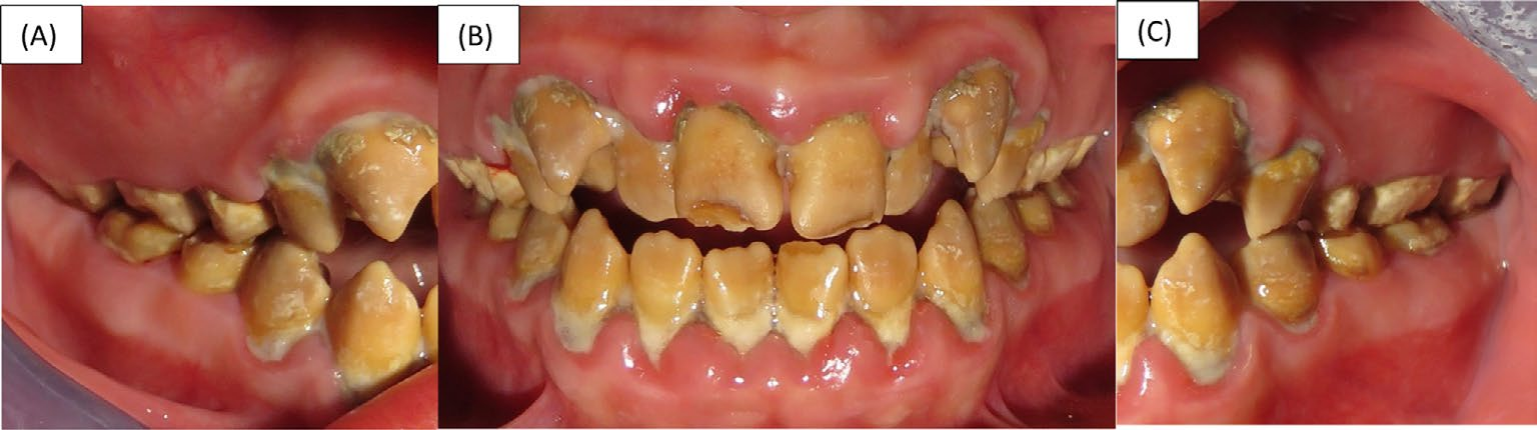
(A–C) Baseline intraoral views revealing heavy sub‐ and supra‐gingival deposits and generalized gingivitis.

Extraoral examination revealed no signs of temporomandibular disorders, a convex facial profile, and competent lips (Figure 1A–C). Intraoral examination showed generalized yellow discoloration with areas of brown pigmentation, plaque accumulation, supra‐ and subgingival calculus, gingival erythema, and bleeding on probing. In addition, anterior open bite, malpositioned maxillary canines, insufficient clinical crown height, asymmetrical gingival contours, and altered vertical parameters were observed (Figure 2A–C). Secondary caries were detected on teeth 11 and 21.

**FIGURE 3:**
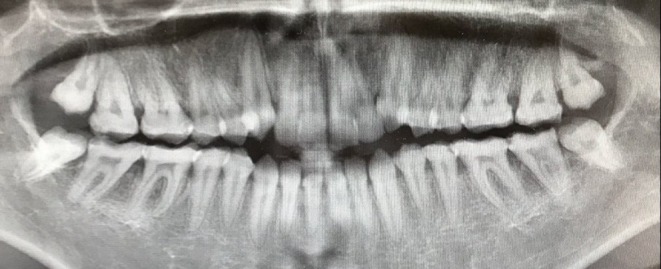
Preoperative orthopantomogram showing almost no enamel surrounding molars.

Orthopantomographic examination revealed minimal enamel coverage around the molars, while pulpal tissues appeared within normal limits (Figure 3).

### 2.3 Diagnosis

The diagnosis of amelogenesis imperfecta had been established during childhood (approximately at the age of 8 years) based on clinical findings and family history. A first‐degree relative (elder sibling) was also diagnosed with amelogenesis imperfecta, supporting a hereditary enamel defect. Clinically, the patient presented with generalized thin enamel, reduced enamel thickness with preserved surface hardness, and the absence of post‐eruptive enamel breakdown, findings consistent with the hypoplastic type of amelogenesis imperfecta. Radiographic examination demonstrated a clear enamel–dentin contrast, further supporting this diagnosis. Other enamel defects, such as molar–incisor hypomineralization and severe dental fluorosis, were considered less likely due to the generalized distribution, early diagnosis, and positive family history.

### 2.4 Treatment Planning (Multidisciplinary Approach)

An orthodontic treatment option was initially proposed to minimize tooth preparation and correct the anterior open bite, with the additional benefit of improved esthetic outcomes. However, the patient expressed a preference for a treatment approach that could be completed in a shorter time frame. Following multidisciplinary consultation, full‐mouth restorative rehabilitation was determined to be the most appropriate treatment option, aiming to improve function and overall dental appearance.

Zirconia crowns were planned for the maxillary anterior teeth (teeth 14, 13, 12, 11, 21, 22, 23, 24) and mandibular anterior teeth (teeth 34, 33, 32, 31, 41, 42, 43, 44), while porcelain‐fused‐to‐metal (PFM) crowns were planned for teeth 15–17, 25–27, 35–37, and 45–47 to reduce treatment costs. Due to insufficient clinical crown height in the posterior regions and the need for open debridement and pocket reduction in the anterior region, a site‐specific crown lengthening approach and extraction of third molars were recommended.

### 2.5 Periodontal Phase

Treatment was initiated with non‐surgical periodontal therapy. Periodontal parameters were assessed using a calibrated periodontal probe. Initial full‐mouth plaque score (FMPS) and full‐mouth bleeding score (FMBS) were recorded as 100% and 82%, respectively. Comprehensive supra‐ and subgingival debridement was performed using ultrasonic devices and hand instruments. Oral hygiene instructions were provided in detail, with emphasis on effective mechanical plaque control.

**FIGURE 4:**
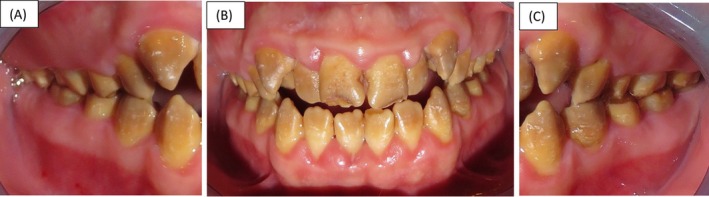
(A–C) Intraoral views after initial non‐surgical periodontal therapy.

Four weeks after initial periodontal therapy, the patient was re‐evaluated, and satisfactory oral hygiene was observed, with both FMPS and FMBS values below 20%. Despite adequate plaque control, residual probing depths of ≥ 5 mm with bleeding on probing persisted in the anterior region, indicating the need for surgical access to achieve periodontal stability prior to prosthetic rehabilitation (Figure 4A–C).

### 2.6 Surgical Phase (Crown Lengthening Procedures)

At this stage, the patient was deemed suitable for surgical periodontal therapy, which was performed in two separate sessions for the maxillary and mandibular arches using the same surgical protocol.

Although the treatment plan included crown lengthening procedures, bone reduction was selectively performed only in the posterior regions where insufficient clinical crown height compromised prosthetic margin placement. In the anterior region, periodontal therapy was limited to gingival leveling and pocket elimination without osseous resection, aiming to preserve supporting bone while establishing stable gingival margins.

**FIGURE 5:**
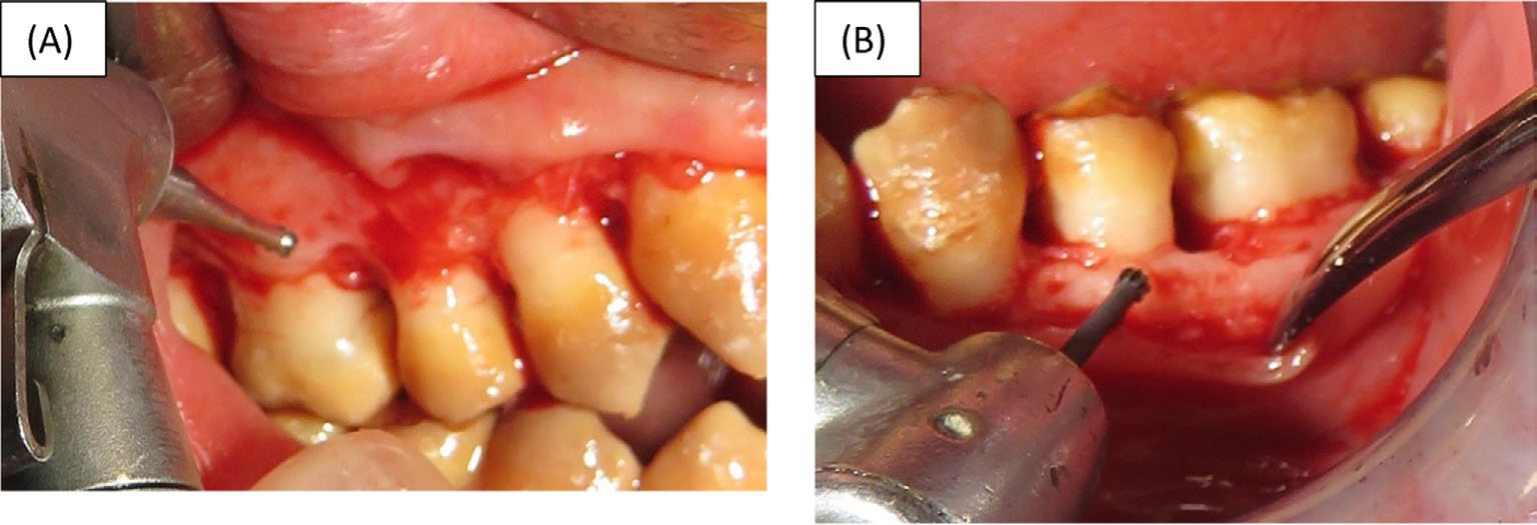
(A, B) Osseous recontouring performed with diamond burs under sterile saline irrigation.

**FIGURE 6:**
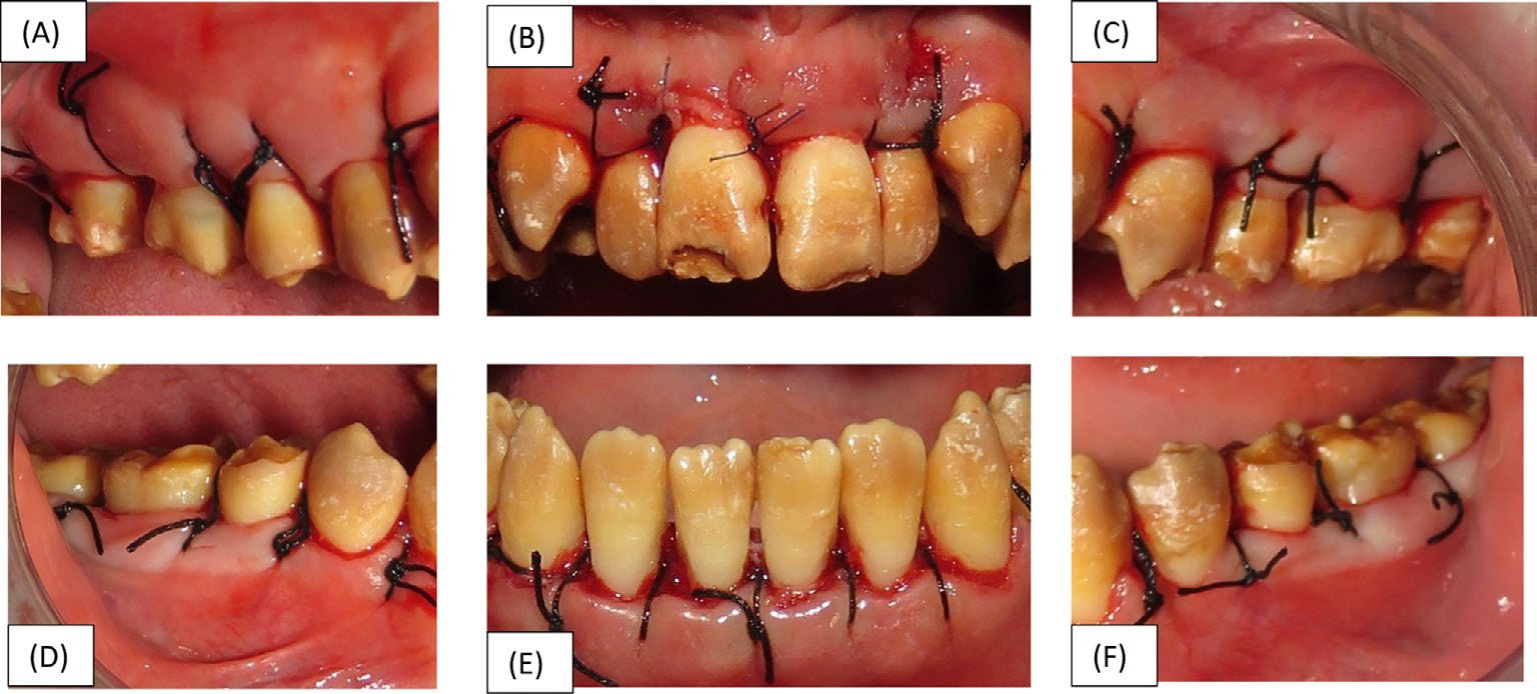
(A–F) Immediate post‐operative view.

Under local infiltrative anesthesia with 4% articaine containing 1:100,000 epinephrine (Ultracaine D‐S Forte, Sanofi), internal bevel and sulcular incisions were performed to remove excess fibrotic tissue. Full‐thickness mucoperiosteal flaps were elevated to ensure adequate visibility and access. Residual granulation tissue was removed using ultrasonic devices and hand instruments. Osseous recontouring was carried out using diamond burs under sterile saline irrigation (Figure 5A,B). Simple interrupted sutures were placed using 3/0 silk sutures (Doğsan, Turkey) (Figure 6A–F).

Postoperative medications included amoxicillin–clavulanic acid, naproxen sodium, and chlorhexidine gluconate mouth rinse.

### 2.7 Postoperative Phase

**FIGURE 7:**
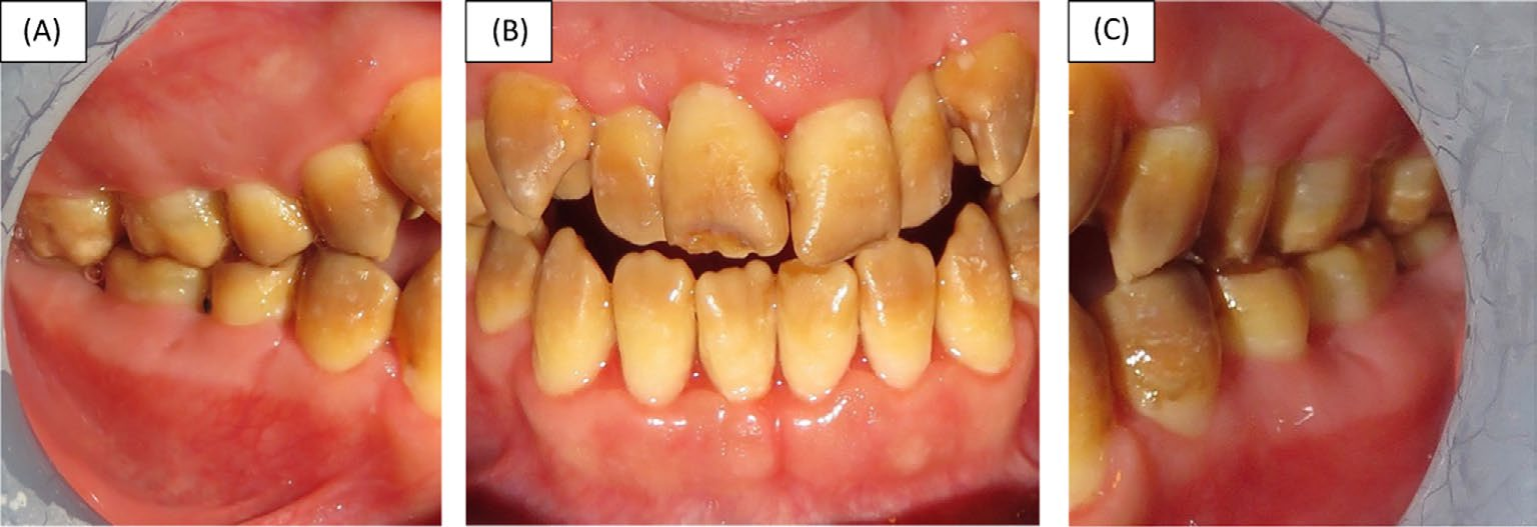
(A–C) Clinical view during the postoperative healing phase following crown lengthening surgery. The image was obtained immediately after professional supragingival debridement performed during a maintenance visit.

The postoperative healing phase was monitored through regular maintenance visits. During this period, professional supragingival debridement was performed as needed to support plaque control and soft tissue healing. Clinical photographs obtained during this phase illustrate the healing process following surgical intervention (Figure 7A–C).

### 2.8 Prosthetic Phase

**FIGURE 8:**
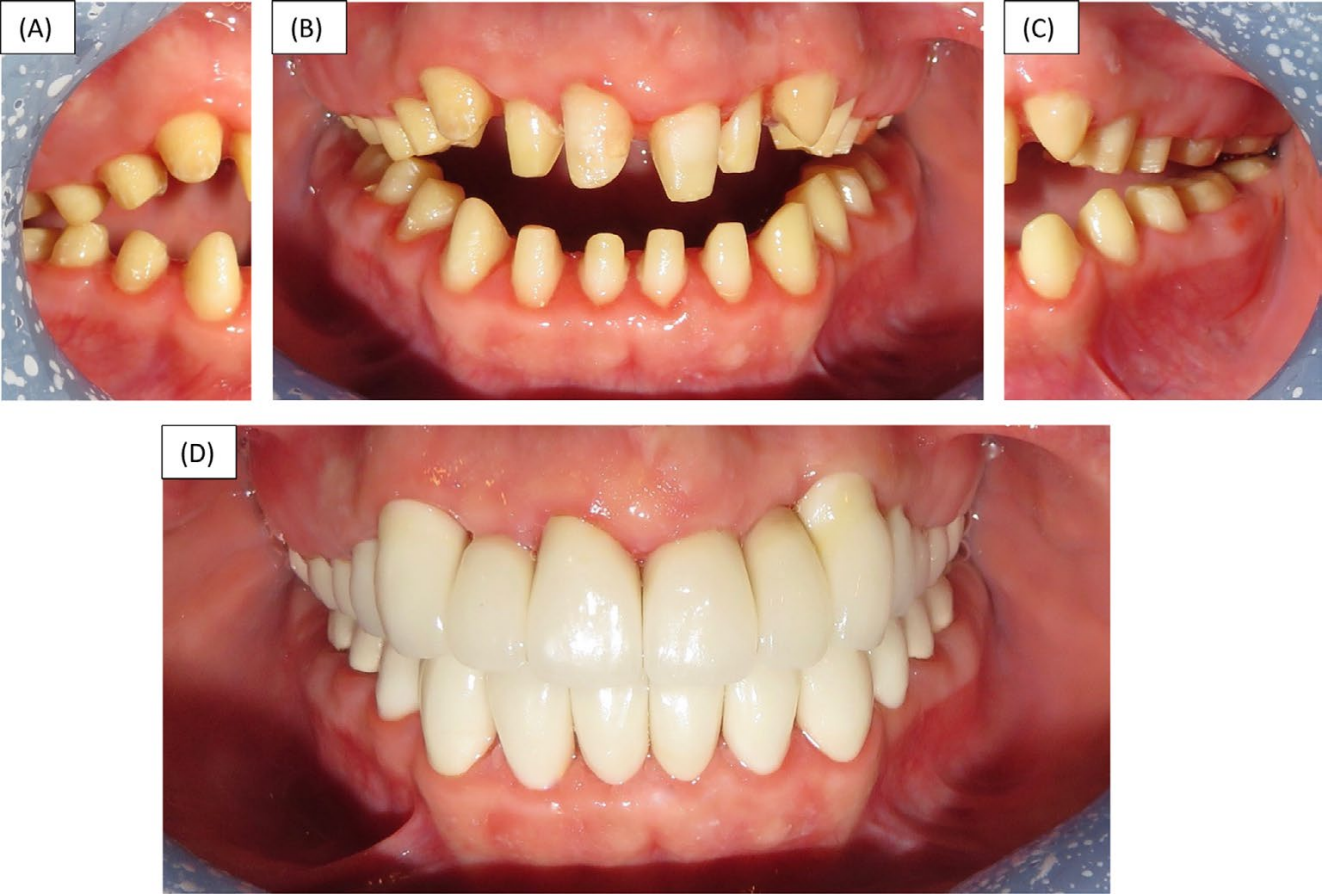
(A–D) Pre‐ and postoperative views of the prosthetic rehabilitation.

Twelve weeks after completion of periodontal therapy, the prosthetic phase was initiated following confirmation of periodontal tissue stability and gingival margin maturation. This healing period was selected in accordance with published periodontal evidence and established clinical practice, allowing resolution of inflammation and stabilization of the periodontal tissues prior to definitive prosthetic rehabilitation. Tooth preparation procedures were then performed, and full‐coverage restorations were fabricated with margin placement adapted to the newly established periodontal architecture. Following cementation of the final restorations, the patient was enrolled in a regular periodontal maintenance program to support long‐term periodontal and prosthetic stability (Figure 8A–D).

### 2.9 Follow‐Up

The patient was followed under a regular periodontal maintenance program for one year, during which satisfactory function and stable periodontal conditions were maintained.

This case report was prepared in accordance with the CARE (CAse REport) guidelines.

## 3 Discussion

Amelogenesis imperfecta (AI) is a rare disease that affects both deciduous and permanent teeth, often resulting in extensive dental needs early in life [16]. In the present case report, a multidisciplinary approach combining comprehensive periodontal and prosthodontic treatment was implemented. Although AI is not considered a progressive disease, teeth with minimal or absent protective enamel are highly susceptible to caries due to dietary habits and bacterial plaque accumulation [12].

Previous guidelines have recommended postponing definitive prosthodontic treatment in children and adolescents with AI and suggested the use of resin composite restorations as a more conservative approach [17, 18]. However, inadequate preservation of the dentition in AI patients may result in severe tooth wear, generalized periodontal inflammation due to plaque accumulation on rough enamel surfaces, and extensive carious lesions requiring endodontic treatment. Ultimately, this may lead to more complex and costly dental interventions [9].

Orthodontic treatment was the first alternative discussed with the patient. In addition to correcting the anterior open bite and arch alignment, forced eruption of the posterior teeth could have increased clinical crown height without bone removal, offering a more conservative preparatory approach before definitive restoration [19]. The patient declined this path because of the time it would require. Direct composite restorations and minimally invasive options—laminate veneers, partial coverage restorations—were also evaluated. The main obstacle here was adhesion: Bonding to AI‐affected enamel is substantially compromised by qualitative changes in enamel composition, with microtensile bond strength reductions of approximately 40% reported relative to sound enamel [20]. Five‐year survival of direct restorations in AI patients is around 50%, and outcomes for minimally invasive indirect restorations bonded to affected enamel remain inconsistent in the literature [20]. Given these limitations and the patient's expressed preference for durable, esthetically stable restorations, full‐coverage crowns were selected.

Clinical evidence consistently demonstrates that indirect restorations—particularly full‐coverage crowns—outperform direct and minimally invasive alternatives in patients with AI in terms of longevity and predictability, largely due to the circumferential removal of irregular AI‐affected enamel and the resulting favorable bonding conditions at the dentin level [20]. Lundgren et al. [21] further demonstrated the superiority of zirconia and glass ceramic crowns over direct resin composites in terms of longevity and soft tissue compatibility. However, achieving predictable full‐coverage rehabilitation requires two prerequisites to be met sequentially: Adequate clinical crown height and established periodontal stability. Residual inflammation and unstable gingival margins would have rendered prosthetic margin placement unreliable and compromised long‐term biological integration of the restorations. The treatment sequence adopted—initial periodontal therapy, site‐specific surgery, structured healing, and definitive rehabilitation—therefore reflects a clinically necessary progression in which each phase was a prerequisite for the next.

One of the most critical factors for the long‐term success of fixed dental prostheses is crown retention, which is directly influenced by occlusocervical dimension, preparation convergence angle, and circumferential morphology [22, 23, 24]. In patients with AI, enamel deficiency frequently results in insufficient clinical crown height, compromising these parameters and necessitating surgical intervention prior to prosthetic rehabilitation. Crown lengthening is a well‐established procedure designed to restore adequate biologic width—defined as the combined dimension of the junctional epithelium and connective tissue attachment coronal to the alveolar bone, ideally approximately 3 mm—and providing sufficient tooth structure for prosthetic margin placement [25, 26, 27, 28, 29, 30, 31, 32]. In the present case, a site‐specific approach was adopted: Open flap debridement without osseous resection was performed anteriorly to eliminate residual pocketing while preserving alveolar bone, whereas selective osseous recontouring was performed in the posterior regions where occlusocervical dimensions were insufficient for predictable crown preparation. This differentiated protocol avoided unnecessary bone reduction at unaffected sites. Lanning et al. [33]. demonstrated that biologic width stabilizes approximately three months postoperatively; accordingly, a healing period of 12 weeks was observed before initiating prosthetic treatment.

Zirconia‐based ceramic crowns were selected for the anterior teeth due to their strength, resistance to masticatory forces, and favorable esthetic properties. Metal‐ceramic restorations were used in the posterior region to reduce treatment cost while maintaining functional durability. Pousette Lundgren et al. [21] reported high clinical success rates for ceramic restorations in teeth affected by AI, supporting the material selection in the present case.

The procedures described in this report are individually well documented in the periodontal and prosthodontic literature. The clinical contribution lies in the explicit account of how they were sequenced and regionally differentiated—the restriction of osseous resection to posterior sites with documented prosthetic insufficiency, the defined stabilization interval prior to tooth preparation, and the requirement for confirmed periodontal stability before prosthetic margin placement. Young adults presenting with the concurrent combination of anterior open bite, generalized periodontal inflammation, and insufficient posterior crown height constitute a subset that has been infrequently reported; the existing literature on amelogenesis imperfecta has largely addressed either pediatric populations or adult patients managed for more isolated clinical problems. This case provides a documented clinical reference for that presentation.

This report has a number of limitations that should be kept in mind. One year of follow‐up is enough to confirm that the initial outcomes held, but it says nothing about restoration longevity or the behavior of the periodontium over a longer horizon. Crown lengthening carries a recognized risk of incomplete tissue adaptation or gingival margin relapse, either of which could affect the marginal fit of the restorations over time. Biologic width was assessed clinically; no histological or radiographic measurement of the bone‐to‐margin distance was obtained. Dentin bonding in AI teeth carries uncertainty even when enamel is bypassed—peritubular thickening and partial tubular obliteration have been reported in affected dentin, and their clinical significance for long‐term cementation is not yet well established. Genetic testing was not performed, so subtype classification rested on clinical and radiographic criteria alone. As with any single case, the outcomes cannot be generalized beyond this patient's presentation.

## 4 Conclusion

Early and comprehensive management may help prevent progressive functional and structural deterioration of the dentition in patients with amelogenesis imperfecta. In the present case, an interdisciplinary treatment approach was associated with improvement in clinical symptoms such as hypersensitivity and gingival bleeding. Crown lengthening was utilized to address insufficient clinical crown height and to facilitate prosthetic rehabilitation under biologically stable conditions. The primary objective of the periodontal intervention was to establish functional and prosthetic stability rather than gingival esthetic optimization. Careful consideration of biologic width remains essential to support periodontal health and to achieve a stable and harmonious emergence profile.

## Author Contributions

**Kubra Burcu Yildirim:** conceptualization, investigation, methodology, writing – original draft, writing – review and editing. **Gizem Ince Kuka:** investigation, resources, writing – review and editing. **Hare Gursoy:** methodology, supervision, writing – review and editing.

## Funding

The authors have nothing to report.

## Ethics Statement

The authors have nothing to report.

## Consent

Written informed consent was obtained from the patient for publication of this case report and accompanying clinical images.

## Conflicts of Interest

The authors declare no conflicts of interest.

## Data Availability

No datasets were generated or analysed during this study. All clinical data are presented within the manuscript.
